# Enhanced Eyeblink Conditioning in Behaviorally Inhibited Individuals is Disrupted by Proactive Interference Following US Alone Pre-exposures

**DOI:** 10.3389/fnbeh.2016.00039

**Published:** 2016-03-10

**Authors:** Michael Todd Allen, Daniel P. Miller

**Affiliations:** ^1^School of Psychological Sciences, University of Northern ColoradoGreeley, CO, USA; ^2^Stress and Motivated Behavior InstituteSyracuse, NY, USA; ^3^Program in Neuroscience, Carthage CollegeKenosha, WI, USA

**Keywords:** proactive interference, behavioral inhibition, associative learning, anxiety, eyeblink conditioning

## Abstract

Anxiety vulnerable individuals exhibit enhanced acquisition of conditioned eyeblinks as well as enhanced proactive interference from conditioned stimulus (CS) or unconditioned stimulus (US) alone pre-exposures (Holloway et al., 2012). US alone pre-exposures disrupt subsequent conditioned response (CR) acquisition to CS-US paired trials as compared to context pre-exposure controls. While Holloway et al. (2012) reported enhanced acquisition in high trait anxiety individuals in the context condition, anxiety vulnerability effects were not reported for the US alone pre-exposure group. It appears from the published data that there were no differences between high and low anxiety individuals in the US alone condition. In the work reported here, we sought to extend the findings of enhanced proactive interference with US alone pre-exposures to determine if the enhanced conditioning was disrupted by proactive interference procedures. We also were interested in the spontaneous eyeblinks during the pre-exposure phase of training. We categorized individuals as anxiety vulnerability or non-vulnerable individuals based scores on the Adult Measure of Behavioral Inhibition (AMBI). Sixty-six participants received 60 trials consisting of 30 US alone or context alone pre-exposures followed by 30 CS-US trials. US alone pre-exposures not only disrupted CR acquisition overall, but behaviorally inhibited (BI) individuals exhibited enhanced proactive interference as compared to non-inhibited (NI) individuals. In addition, US alone pre-exposures disrupted the enhanced acquisition observed in BI individuals as compared to NI individuals following context alone pre-exposures. Differences were also found in rates of spontaneous eyeblinks between BI and NI individuals during context pre-exposure. Our findings will be discussed in the light of the neural substrates of eyeblink conditioning as well as possible factors such as hypervigilance in the amygdala and hippocampal systems, and possible learned helplessness. Applications of these findings of enhanced proactive interference in BI individuals to pre-exposure therapies to reduce anxiety disorders such as posttraumatic stress disorder (PTSD) will be discussed.

## Introduction

Recent work has focused on a learning diathesis model of anxiety disorders involving anxiety vulnerability and associative learning (Allen et al., 2014, 2016; Myers et al., 2012; Caulfield et al., 2013; Holloway et al., 2014). In these studies, anxiety vulnerability has been operationally defined based on personality factors such as trait anxiety and behavioral inhibition (BI).

Trait anxiety has been measured by the State Trait Anxiety Inventory or STAI (Spielberger et al., 1983) The STAI is a self-report inventory which consists of 20 items that address state anxiety and 20 items that address trait anxiety, which distinguishes between those who experience anxiety as a characteristic of their personality (i.e., trait anxiety) compared to situational anxiety (i.e., state anxiety). Previously Holloway et al. (2012), operationally defined trait anxious individuals as those with scores in the top one third of the distribution of the trait anxiety scores.

BI has been measured by the Adult Measure of Behavioral Inhibition (AMBI; Gladstone and Parker, 2005). The AMBI is a self-report inventory which consists of 16 items that address avoidance and other behaviors that have been linked to anxiety vulnerability. BI is defined as a temperamental tendency to withdraw from or avoid novel social and non-social situations (Kagan et al., 1987; Morgan, 2006). In addition to avoidance, BI also includes social reticence and enhanced reactivity to novelty, threat, and uncertainty (Hirshfeld et al., 1992; Schwartz et al., 2003a,b). It has long been considered a vulnerability factor for the development of anxiety-related disorders including posttraumatic stress disorder (PTSD; North et al., 2008). This reserved response, and in extreme cases of inactivity, may be linked to trait anxiety. However, BI is merely a risk factor, not a cause for a wide variety of anxiety disorders. Indeed, a diathesis model approach to stress has been used to conceptualize the combination of individual vulnerabilities (e.g., BI) with exposure to environmental stressors, with the outcome being the development of pathology (e.g., Mineka and Zinbarg, 2006; Servatius et al., 2008). Previously, Holloway et al. (2014) operationally defined behaviorally inhibited individuals as those who scored in the top on third of the distribution of AMBI scores. More recently, Allen et al. (2014) defined BI based on a median split of the AMBI scores.

Anxiety vulnerability has been found to be linked to enhanced associative learning in classical eyeblink conditioning in humans. Eyeblink conditioning involves the pairing of a conditioned stimulus (CS) tone with an unconditional stimulus (US) corneal air puff which results in learning a conditioned response (CR) eyeblink to the previously neutral CS. Partial reinforcement with either CS or US alone trials inter-mixed with CS-US paired trials produced enhanced acquisition of conditioned responses in anxiety vulnerable individuals that was greater than that observed with 100% CS-US paired trials (Allen et al., 2014). Extending and varying the time between trials [i.e., the inter-trial interval (ITI)] also enhanced acquisition of conditioned responses in anxiety vulnerable individuals (Allen et al., 2016). These eyeblink conditioning protocols share a level of uncertainty about stimulus and trial delivery.

Another learning protocol that involves some degree of uncertainty that has also been found to be enhanced by anxiety vulnerability is proactive interference following stimulus pre-exposures. Pre-exposure to the individual conditioning stimuli (CS tones or US air puffs) prior to paired CS-US training results in slower acquisition of CR. Holloway et al. (2012) assessed conditioned eyeblink response acquisition after equal numbers of CS, US, and explicitly unpaired CS and US pre-exposures prior to standard delay tone- air puff training. Proactive interference was most evident in the US pre-exposure group relative to context pre-exposure controls. This US pre-exposure effect was stronger than the more well known CS pre-exposure effect known as latent inhibition (LI). High trait anxious individuals were found to have enhanced overall acquisition as well as greater proactive interference relative to non-vulnerable individuals (Holloway et al., 2012).

However, some aspects of the analyses reported by Holloway et al. (2012) bring into question some of their conclusions. It appears from visual examination of published figures that the differences in proactive interference between high and low trait anxiety individuals may be due to higher rates of CR acquisition by the high trait anxiety group in the context pre-exposure condition rather than lower rates of CR acquisition in the stimulus pre-exposure condition. It is therefore unclear whether the reported enhanced proactive interference in the high trait anxiety group was an artifact of enhanced CR acquisition by the high trait anxiety individuals in the control condition. In addition, enhanced acquisition for high trait anxious individuals was reported for the context pre-exposure condition, but not for the stimulus pre-exposure conditions. Based on visual examination of the published figures, the high and low trait anxious individuals do not appear to differ significantly in the US alone pre-exposure condition. It appears that stimulus pre-exposure eliminated the enhanced acquisition observed in anxiety vulnerable individuals. Analyses of these effects were not reported by Holloway et al. (2012).

The reporting of measures of anxiety vulnerability was also incomplete. Holloway et al. (2012) collected data on trait anxiety and BI. However, while results on anxiety vulnerability effects were reported based on trait anxiety, the effects of BI on eyeblink conditioning and proactive interference were not mentioned. The effects of BI on proactive interference are of particular interest based on its effectiveness in categorizing anxiety vulnerable and non-vulnerable which has identified enhanced associative learning in recent eyeblink studies (Allen et al., 2014, 2016; Holloway et al., 2014).

Holloway et al. (2012) also tested some non-associative factors such as reactivity to the US alone air puff that could account for differences in associative learning between anxiety vulnerable and non-vulnerable individuals. There were no differences in unconditioned response (UR) amplitude between the vulnerable and non-vulnerable groups. In addition, there were no sensory habituation effects on the UR amplitude across the US pre-exposure phase. However, spontaneous eyeblink rates during the pre-exposure phase were not reported. It is possible that anxiety vulnerable individuals differ in their rate of blinking which could in turn be a factor in the enhanced acquisition of eyeblinks to the CS.

Based on these unanswered questions, we wanted to extend the US pre-exposure effect from the Holloway et al. (2012) study with special attention to these issues. We selected US alone pre-exposure for this study since it was the proactive interference protocol that had the greatest disruptive effect on subsequent CR acquisition. We also utilized a different measure of anxiety vulnerability than that reported by Holloway et al. (2012). They categorized participants such that those with trait anxiety scores in the top one third of the distribution were labeled as anxiety vulnerable and those in the bottom two thirds were labeled non-vulnerable. In the current study, we utilized AMBI scores as the measure of anxiety vulnerability with participants categorized as anxiety vulnerable and non-vulnerable based on a median split. Recent studies suggest that BI is a stronger measure of anxiety vulnerability than trait anxiety (Allen et al., 2014; Holloway et al., 2014). We planned direct comparisons between anxiety vulnerable and non-vulnerable individuals within the context alone and US alone pre-exposure conditions. In addition, we also monitored spontaneous blinks during the pre-exposure phase to determine if there were non-associative differences in anxiety vulnerable and non-vulnerable individuals that could account for differences in associative learning.

We hypothesized that US alone pre-exposures would disrupt subsequent CR acquisition as compared to context alone pre-exposure. We also hypothesized that while enhanced acquisition would be evident in BI individuals in the context (no stimulus) pre-exposure condition, there would be no BI enhancement effect in the US alone pre-exposure condition. We also hypothesized that there would be no significant differences in spontaneous blinking during context and US alone pre-exposures between anxiety vulnerable and non-vulnerable individuals.

## Materials and Methods

### Participants

Sixty-six college-aged students were recruited from the University of Northern Colorado, School of Psychological Sciences. Students voluntarily participated to receive class credit or extra credit for psychology coursework. Our sample consisted of 45 females and 21 males with a mean age of 22.4 years (SD = 8.4, range 18–55) and mean education of 13.7 years (SD = 1.5, range 12–16). Informed consent was obtained in accordance with procedures approved by the University of Northern Colorado Institutional Review Board.

### Apparatus

The eyeblink conditioning apparatus and procedures were similar to that previously described (Beck et al., 2008). The tone stimulus was produced with Coulbourn Instruments (Allentown, PA, USA) signal generators and passed to a David Clark aviation headset (Model H10–50, Worchester, MA, USA). Sound levels were verified with a Realistic sound meter (RadioShack, Fort Worth, TX, USA). The headset was fitted with a boom placed 1 cm from the cornea that delivered a 5 psi air puff US via sylastic tubing connected to a regulator and released by a computer controlled solenoid valve (Clipper Instruments, Cincinnati, OH, USA). To record the eyelid electromyographic (EMG) signal, pediatric silver/silver chloride EMG electrodes with solid gel were placed above and below the right eye, with the ground electrode placed on the neck. The EMG signal was passed to a medically isolated physiological amplifier (UFI, Morro Bay, CA, USA), low-pass filtered and amplified 10K. The EMG signal was sampled at 500 Hz by an A/D board (PCI 6025E, National Instruments, Austin, TX, USA) connected to an IBM-compatible computer. Software control of stimulus generation was performed by LabView (National Instruments).

### Psychometric Scales

Study participants completed the AMBI (Gladstone and Parker, 2005). The AMBI is a 16-item self-report inventory that assesses current tendency to respond to new stimuli with inhibition and/or avoidance, and has also been shown to be a measure of anxiety proneness.

### BI Groups

Participants were divided into behaviorally inhibited (BI) and non-inhibited (NI) groups based on a median split of AMBI scores. This methodology was based on previous eyeblink conditioning studies with BI (Caulfield et al., 2013; Allen et al., 2014) and allowed for comparable sample sizes in our BI and NI groups.

### Conditioning Session

Upon arrival to the study, participants provided informed consent and were instructed that the study was going to evaluate responses to tones and air puffs to the eye, that they were to watch a video (e.g., a nature video with sound muted), and that they were to remain awake during the testing session. Participants were then fitted with EMG electrodes and headphones, EMG signal quality was verified, and the conditioning program was started. Participants received either US alone or context alone (i.e., no stimuli) pre-exposures for 30 trials, followed immediately by 30 CS-US paired trials. Paired CS-US trials included a 500 ms/1200 Hz pure tone CS overlapping and co-terminating with the 50 ms US air puff. The ITI varied pseudo-randomly between 30 ± 5 s.

### Signal Processing and Data Reduction

EMG data were evaluated on a trial-by-trial basis for all participants. Processing of eyeblink responses followed methods previously reported (Beck et al., 2008). To determine the occurrence of an eyeblink, EMG activity was first lowpass filtered with a Lowess filter (Stat-Sci, Tacoma, WA, USA) using a time constant of 0.025, and a smoothing interval of 5. For an eyeblink response to be scored, smoothed EMG activity in a 500-ms window beginning at the onset of the CS had to exceed the mean activity, plus four times the standard deviation, of the activity in a 125-ms comparator window that immediately preceded the CS window. An alpha (orienting) response was defined as an eyeblink occurring within 80 ms of CS onset, and this trial was not counted as a CR. A CR was defined as an eyeblink occurring 80 ms after CS onset but before US onset. A UR was scored when an eyeblink was produced 0–100 ms after US onset.

Those sessions with excessive signal noise (loss of more than 10% of trials due to alpha responses or no UR detected), equipment malfunction, or incomplete session data (e.g., falling asleep or loss of UR), were discarded and not used for further analysis. In addition, the data of participants who had excessive levels of spontaneous blinks or alpha responses (80% or more CRs starting in the first block) were excluded from data analysis.

### Data Analysis

To examine the main effects and interactions of anxiety vulnerability and CR acquisition, the 60 trial conditioning session was divided into 10 trial blocks and evaluated independently for 30 pre-exposure trials (3 blocks) and 30 acquisition trials (3 blocks). Between group measures included pre-exposure (context alone vs. US alone), and BI (behaviorally inhibited vs. non-inhibited), with Block as a within subject measure. Pairwise comparisons were planned between the high and low BI groups as well as between the two pre-exposure conditions. The level of significance was set at *p* < 0.05.

## Results

Inspection of the eyeblink data resulted in rejection of data from 28 participants (17 females and 11 males). Most exclusions were due to excessive spontaneous blinking or alpha responses from the start of conditioning. Psychometric and demographic data for the remaining 38 participants including the BI and NI groups for the context and US alone pre-exposure conditions are summarized in Table 1. BI and NI individuals were categorized based on a median split at an AMBI score of 13.5. This median score is comparable to previous work in which the median scores ranged from 11.5 to 14.5 (Allen et al., 2014).

**Table 1:** **Demographic and psychometric data of context and US pre-exposure groups**.

Pre-exposure condition	Behavioral inhibition level	*n* (male)	AMBI (se)	Mean % CR Acquisition (se)
Context pre-exposure	Non-inhibited	11 (2)	10.8 (0.5)	44.5 (5.4)
	Inhibited	9 (6)	18.2 (2.1)	59.3 (4.1)
US alone pre-exposure	Non-inhibited	9 (1)	7.5 (0.5)	46.7 (7.8)
	Inhibited	9 (6)	20.1 (2.1)	32.6 (5.5)

### Effects During Pre-Exposure

**Figure 1:**
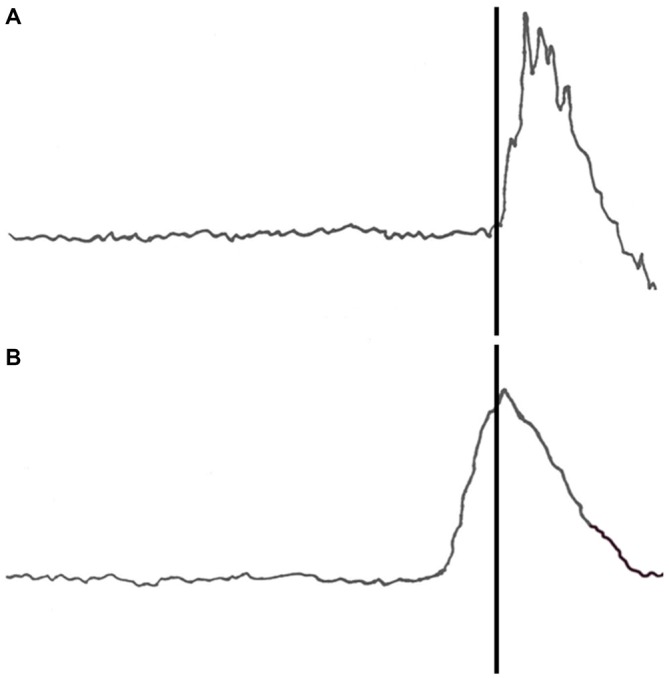
**Recorded eyeblink responses from a US alone pre-exposure trial (upper panel) and a CS-US paired trial (lower panel).** The eyeblink responses are filtered eyelid electromyographic (EMG) signals. The vertical line indicates the onset of the US air puff. **(A)** The upper panel represents an unconditioned response (UR) to the air puff on a US alone pre-exposure trial. **(B)** The lower panel represents a well-timed conditioned response (CR) on a tone-air puff training trial.

Eyeblinks were monitored during the 450 ms window prior to the time of the US presentation during which the CS would occur in the CS-US paired trials. No stimuli were presented during this window in either the US alone or context alone pre-exposure trials. There was no main effect of US pre-exposure (*p* = 0.254) or BI (*p* = 0.46) for eyeblinks recorded during the pre-exposure phase. There was a significant interaction of BI × block for the spontaneous blink rate during context pre-exposure trials such that the high BI group had higher initial blinking than the NI group and that was reduced as context pre-exposure continued (*F*_(1,34)_ = 3.509, *p* < 0.05). No other main effects or significant interactions were found in context or US pre-exposure conditions. An example of an unconditioned response (UR) on an air puff alone pre-exposure trial is shown in Figure 1A.

### Effects During Acquisition

**Figure 2:**
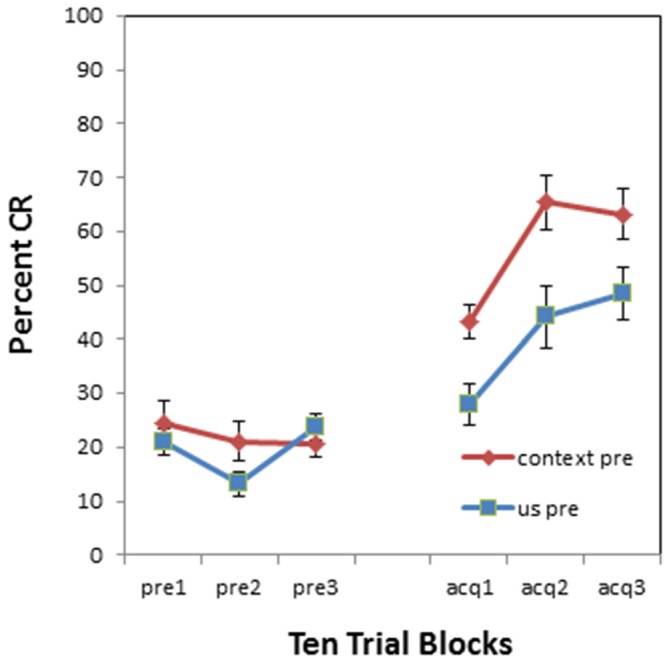
**Pre-exposure and acquisition phases of eyeblink conditioning for groups receiving context alone or US alone pre-exposure.** The pre-exposure (pre) phase consisted of 30 trials (context alone or US alone presentations) while the acquisition (acq) phase consisted of 30 CS-US paired trials. There were no significant effects on spontaneous blinks during the pre-exposure phase. All participants acquired conditioned eyeblinks during acquisition training, but US alone pre-exposures disrupted CR acquisition as compared to context pre-exposures. Error bars represent standard error of the mean.

All participants acquired CRs. An example of a conditioned response to a paired tone and air puff acquisition trial is shown in Figure 1B. Three blocks of CS-US paired trials produced a significant increase in CR following both pre-exposure conditions. This finding was supported by a main effect of block (*F*_(1,68)_ = 24.383, *p* < 0.001) as shown in Figure 2. There were no significant interactions of block × BI (*p* = 0.486), block × pre-exposure (*p* = 0.226) or block × BI × pre-exposure (*p* = 0.166).

Pre-exposure of the US alone produced a proactive interference effect such that individuals pre-exposed to the US alone exhibited fewer CRs than individuals pre-exposed to the context only. This finding was supported by a main effect of pre-exposure (*F*_(1,34)_ = 14.864, *p* < 0.001) as shown in Figure 2.

**Figure 3:**
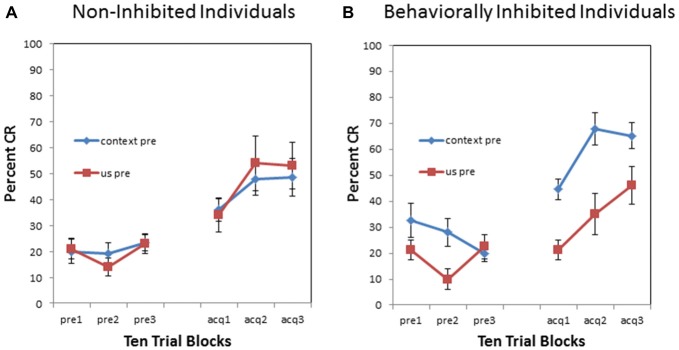
**Pre-exposure and acquisition phases of eyeblink conditioning for non-inhibited (NI) individuals (left panel) and behaviorally (BI) individuals (right panel) receiving context alone (no stimulus) pre-exposures or US alone pre-exposure. (A)** NI individuals did not exhibit any significant differences in pre-exposure or acquisition between context and US alone pre-exposures. **(B)** Behaviorally inhibited (BI) individuals exhibited proactive interference or slowed CR acquisition following US alone pre-exposures. BI individuals also initially exhibited more spontaneous eyeblinks during context (no stimulus) pre-exposures which habituated as pre-exposure trials progressed. Error bars represent standard error of the mean.

There was also a significant pre-exposure × BI interaction (*F*_(1,34)_ = 5.460, *p* < 0.05). This interaction is evident in Figure 3 which compares context vs. US pre-exposure separately for NI individuals and BI individuals. There was a significant proactive interference effect evident in the BI individuals (*F*_(1,18)_ = 34.134, *p* < 0.001) as shown in Figure 3B. However, NI individuals did not exhibit a proactive interference effect (*F*_(1,16)_ = 0.581, *p* = 0.457) as shown in Figure 3A. There was no significant interaction between block and pre-exposure for the NI individuals (*p* > 0.90) or BI individuals (*p* > 0.010).

**Figure 4:**
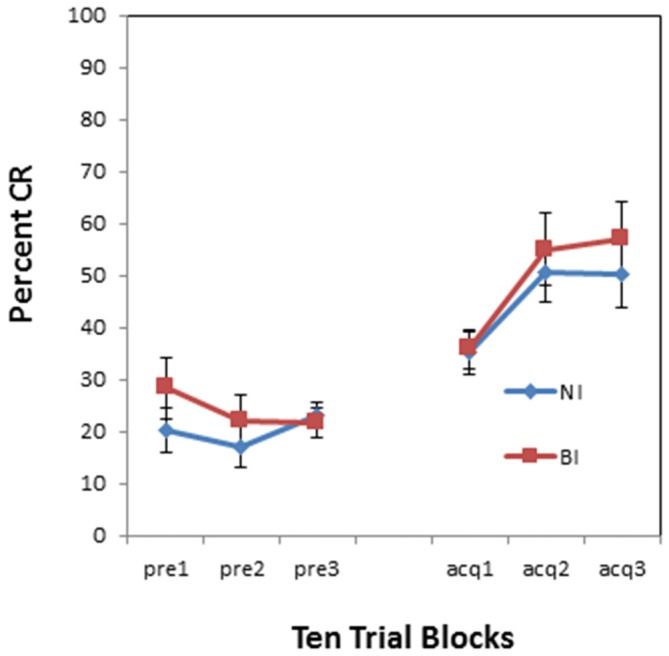
**Pre-exposure and acquisition phases of eyeblink conditioning for NI and BI individuals.** There were no significant differences between NI and BI individuals in spontaneous blinks during the pre-exposure phase and conditioned eyeblinks during the acquisition phase. Error bars represent standard error of the mean.

**Figure 5:**
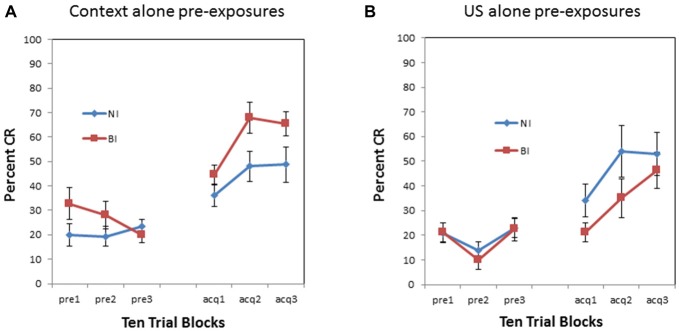
**Pre-exposure and acquisition phases of eyeblink conditioning for NI individuals and BI individuals receiving context alone (no stimulus) pre-exposures (left panel) or US alone pre-exposures (right panel). (A)** BI individuals exhibited enhanced acquisition of CRs following context alone pre-exposures as compared to NI individuals.** (B)** Enhanced acquisition of CRs was not evident in anxiety vulnerable individuals following US alone pre-exposures as compared to non-vulnerable individuals. There was a non-significant trend for BI individuals to express fewer CRs than NI individuals following US alone pre-exposures. Error bars represent standard error of the mean.

There was no overall effect of BI (*F*_(1,34)_ = 0.001, *p* = 0.974) as shown in Figure 4. However, there was a pre-exposure by BI interaction (*F*_(1,34)_ = 5.460, *p* < 0.05). This interaction is evident in Figure 5 which compares NI and BI individuals separately for the context alone and US alone pre-exposure conditions. BI individuals exhibited more CRs than NI individuals in the context pre-exposure condition (*F*_(1,18)_ = 4.976, *p* < 0.05) as shown in Figure 5A. However there was a non-significant trend for BI individuals to exhibit less CRs than NI individuals in the US pre-exposure condition (*F*_(1,16)_ = 2.438, *p* = 0.138) as shown in Figure 5B. There were no significant interactions between block and BI group for acquisition following the context (*p* = 0.383) and US air puff alone conditions (*p* = 0.75).

## Discussion

The current study sought to further explore the effects of US alone pre-exposure on anxiety vulnerable and non-vulnerable individuals. Overall, we replicated the finding of Holloway et al. (2012) of enhanced proactive interference in anxiety vulnerable individuals as compared to non-vulnerable individuals. Our study extended the study of anxiety vulnerability and proactive interference in that we used BI as our measure of anxiety vulnerability rather than trait anxiety. Our grouping of individuals as anxiety vulnerable and non-vulnerable also differed from the methodology of Holloway et al. (2012) which compared the top one third and bottom two thirds of the distribution of trait score. The current methodology of a median split provided for equal sample sizes.

Our result also furthered the findings of Holloway et al. (2012) in that we observed a significant proactive interference effect in the anxiety vulnerable, but not the non-vulnerable, individuals. It seems that BI individuals are overly sensitive to US pre-exposures as compared to NI individuals. Visual analysis of the data indicates that the proactive interference effect is so strong in BI individuals that by the end of acquisition, conditioned responding by BI individuals has achieved the level of initial conditioned responding in NI individuals. Proactive interference is the first eyeblink conditioning protocol which has been found to disrupt the enhancement observed in BI individuals.

This difference in proactive interference between anxiety vulnerable and non-vulnerable individuals is of interest given the previous reports of difficulty in producing proactive interference effects with CS alone pre-exposures (i.e., latent inhibition, LI) in human eyeblink conditioning (Allen et al., 2002b; Holloway et al., 2012). Difficulties in producing proactive interference effects may be due in some part to differences in anxiety vulnerability that were not accounted for in these previous human eyeblink studies.

Prior work with anxiety vulnerable individuals has found enhanced acquisition of conditioned eyeblinks (Allen et al., 2014; Holloway et al., 2014). In the current work, we found that anxiety vulnerable individuals demonstrated enhanced eyeblink conditioning following the context pre-exposures, but not the US alone pre-exposures. CR acquisition for BI individuals pre-exposed to the US air puff alone did not differ from NI individuals. The BI individuals’ learning curves appeared to actually be lower than NI individuals. This findings is similar to the graphs of Holloway et al. (2012), but these analyses directly comparing BI and NI individuals with US alone pre-exposures were not reported in that study. Our findings extend those of Holloway et al. (2012) to indicate that US alone pre-exposures wipe out the enhancement of conditioned eyeblink responses observed following context alone pre-exposures in anxiety vulnerable individuals.

It is of interest that there were some differences in spontaneous blinking in BI individuals. Some aspects of CR facilitation may be due to differences in spontaneous blink rates or responsivity to US alone trials. Recent studies found no differences in the amplitude of URs prior to conditioning (Holloway et al., 2012, 2014; Allen et al., 2014). However, the current findings indicate that BI individuals exhibit more spontaneous blinks, which if replicable, could be of significant clinical importance.

The current findings of enhanced proactive interference and a disruption of enhanced CR acquisition in BI individuals can be explained based on the known neural substrates of eyeblink conditioning, specifically in studies involving proactive interference. Cerebellar and brainstem circuits are known to underlie acquisition, retention, and extinction of eyeblink conditioning across several mammalian species including rabbits, rodents, and humans (for review, see Thompson and Steinmetz, 2009). In delay conditioning, CS and US partially overlap and co-terminate. This form of eyeblink conditioning, utilized in the current, is known to require the cerebellum and brainstem, but not other brain structures such as the hippocampus (Schmaltz and Theios, 1972; Gabrieli et al., 1995), motor cortex (Ivkovich and Thompson, 1997) or the entire cerebral cortex (Mauk and Thompson, 1987). The hippocampus (Solomon et al., 1986; Moyer et al., 1990, 2015) and related cortical areas such as the medial prefrontal cortex (Kronforst-Collins and Disterhoft, 1998; Weible et al., 2000) and retrosplenial cortex (Weible et al., 2000) have been found to be necessary for trace eyeblink conditioning in which there is a stimulus free period between the CS and US, but these brain areas are not required in delay conditioning as examined in the current study. However, strong evidence also exists that the associative learning in the cerebellum during delay conditioning can be modified by septo-hippocampal (Berry and Thompson, 1979; Allen et al., 2002c) and amygdala inputs (Whalen and Kapp, 1991; Weisz et al., 1992; Blankenship et al., 2005).

Prior eyeblink conditioning studies in rabbits have identified the neural substrates for the proactive interference tasks involving CS alone pre-exposures known as LI and uncorrelated CS and US pre-exposures known as learned irrelevance (LIRR). The neural substrates of LI were first identified as the hippocampus with non-selective lesions (Solomon and Moore, 1975), and more recently as in the entorhinal cortex rather than the hippocampus with selective ibotenic acid lesions for LI (Shohamy et al., 2000) and LIRR (Allen et al., 2002a). The current proactive interference effect with US alone pre-exposures has not been tested with lesion studies in rabbits.

Prior studies of BI in humans have hypothesized the BI enhancement effect comes about through limbic modulation of the cerebellum from the hippocampus and amygdala (Allen et al., 2014). The hippocampus, but not the amygdala, has a long history of involvement in pre-exposure effects like LI with CS alone pre-exposure and LIRR with uncorrelated pre-exposures of the CS and US.

Computational models and theories have proposed that pre-exposure effects come about through some mechanism of compression or fusion of the unpaired stimuli and the background context (Bunsey and Eichenbaum, 1993; Myers et al., 1995). Compression or fusion of the individual stimulus, in this case the US, with the background context slows subsequent learning of the CS-US association. When CS-US training begins, this representation of the stimulus must first be uncompressed from the background context so that it can then be associated with the US for the formation of CRs. The US air puffs pre-exposed alone should be compressed into the background context in a fashion similar to the CS tones in LI to produce the subsequent slowing of CR acquisition to CS-US paired trials.

However, in the current work, BI individuals differed from NI individuals in response to the US alone pre-exposures as compared to the context alone pre-exposures. BI, but not NI individuals expressed proactive interference following US alone pre-exposures. BI individuals may experience hypervigilance to uncertain aversive events such as unpredicted air puffs. Therefore, if more attention is paid by BI individuals to the US alone pre-exposures, they may undergo stronger compression or fusion of this stimulus with the background context, thus taking longer to form the CS-US association necessary to produce conditioned eyeblinks to the CS tone.

Another possible mechanism for enhanced proactive interference in BI individuals could be heightened associative learning through compression in the entorhinal system. It would be of interest to test the effects of BI or anxiety vulnerability in other learning paradigms such as sensory preconditioning or negative patterning that have been hypothesized as involving entorhinal compression (Myers et al., 1995).

Another possible mechanism for this enhanced proactive interference effect comes from the Wistar Kyoto (WKY) inbred rat strain. WKY rats are a BI strain that has been suggested to be a model of stress and anxiety vulnerability. WKYs emit more CRs and achieve higher asymptotic performance during classical eyeblink conditioning than the outbred Sprague Dawley (SD) strain (Ricart et al., 2011). These findings fit with the basic findings in classical conditioning of anxiety vulnerable individuals (Allen et al., 2014; Holloway et al., 2014). However, proactive interference in eyeblink conditioning was less evident in WKY rats than in SD controls with CS alone or US alone pre-exposures (Ricart et al., 2011). However, WKYs have been found to exhibit enhanced learned helplessness in other learning paradigms (Paré, 1989, 1992, 1994). The lack of proactive interference in WKY rats was theorized by Ricart et al. (2011) to be due to hypervigilance and an inability to disengage from discrete environmental stimuli. Another possible explanation is that WKY rats exhibit learned helplessness to US alone pre-exposures. A significant difference between eyeblink conditioning in humans and rats is that the US in rats is an electric shock to the eye rather than a corneal air puff. The differences in the aversive nature of an electric shock and a corneal air puff could explain these differences between findings in humans and rats.

The major finding of the current work is that anxiety vulnerable individuals appear more sensitive to pre-exposure effects like proactive interference than non-vulnerable individuals. This proactive interference is so strong in BI individuals as to disrupt the enhanced acquisition of conditioned eyeblink observed with BI individuals pre-exposed to the context alone. Future work should test whether PTSD sufferers exhibit similar effects with US alone pre-exposures and proactive interference.

PTSD has been hypothesized to occur through enhanced associative learning (Pitman, 1988; Pitman et al., 2000; Myers et al., 2012). Prior work with eyeblink conditioning has found PTSD patients exhibit enhanced associative learning (Myers et al., 2012). The problems experienced by PTSD sufferers following trauma exposure can be understood as a learned response to some event or signal that predicts the traumatic event. For example, fear of loud noises or crowds (i.e., the CS) that predicted a previous traumatic event such as an explosion (i.e., the US). Based on the current findings, pre-exposure to traumatic events could disrupt the formation of associations between neutral environmental events and traumatic events and thus limit the formation of PTSD symptoms to these neutral stimuli. Currently, a major form of therapy for anxiety disorders like PTSD is exposure therapy. Based on the current findings, pre-exposure therapy may also be a possible preemptive measure that would limit formation of PTSD symptoms.

## Author Contributions

MTA and DM were involved in the design of the study. MTA collected the data and prepared the manuscript. MTA and DM were involved in the revisions to the manuscript.

## Funding

Funding was provided by the University of Northern Colorado and the Stress and Motivated Behavior Institute.

## Conflict of Interest Statement

The authors declare that the research was conducted in the absence of any commercial or financial relationships that could be construed as a potential conflict of interest.
